# 60/30: 60% of the Morbidity-Associated Multiple Sclerosis Disease Burden Comes From the 30% of Persons With Higher Impairments

**DOI:** 10.3389/fneur.2020.00156

**Published:** 2020-03-06

**Authors:** Marco Kaufmann, Milo Alan Puhan, Anke Salmen, Christian P. Kamm, Zina-Mary Manjaly, Pasquale Calabrese, Sven Schippling, Stefanie Müller, Jens Kuhle, Caroline Pot, Claudio Gobbi, Nina Steinemann, Viktor von Wyl, Swiss Multiple Sclerosis Registry (SMSR)

**Affiliations:** ^1^Department of Epidemiology, Epidemiology, Biostatistics and Prevention Institute, University of Zurich, Zurich, Switzerland; ^2^Department of Neurology, Inselspital, University Hospital Bern and University of Bern, Bern, Switzerland; ^3^Neurology and Neurorehabilitation Centre, Luzerner Kantonsspital, Lucerne, Switzerland; ^4^Department of Neurology, Schulthess Clinic, Zurich, Switzerland; ^5^Department of Health Sciences and Technology, ETH Zurich, Zurich, Switzerland; ^6^Division of Molecular and Cognitive Neuroscience, University of Basel, Basel, Switzerland; ^7^Neuroimmunology and Multiple Sclerosis Research, Department of Neurology, University Hospital Zurich and University of Zurich, Zurich, Switzerland; ^8^Center for Neuroscience Zurich, University of Zurich and Federal Institute of Technology (ETH) Zurich, Zurich, Switzerland; ^9^Department of Neurology, Cantonal Hospital St. Gallen, St. Gallen, Switzerland; ^10^Neurologic Clinic and Policlinic, Departments of Medicine, Biomedicine and Clinical Research, University Hospital and University of Basel, Basel, Switzerland; ^11^Service of Neurology, Department of Clinical Neurosciences, Lausanne University Hospital and University of Lausanne, Lausanne, Switzerland; ^12^Faculty of Biomedical Sciences, Università della Svizzera Italiana (USI), Lugano, Switzerland; ^13^Department of Neurology, Multiple Sclerosis Center (MSC), Neurocenter of Southern Switzerland, Lugano, Switzerland

**Keywords:** burden, DALY, morbidity, mortality, SMSR, epidemiology

## Abstract

**Background:** Multiple sclerosis (MS) is the most common chronic, non-traumatic, neurologic disease in young adults. While approximate values of the disease burden of MS are known, individual drivers are unknown.

**Objective:** To estimate the age-, sex-, and disease severity-specific contributions to the disease burden of MS.

**Methods:** We estimated the disease burden of MS using disability-adjusted life years (DALYs) following the Global Burden of Disease study (GBD) methodology. The data sources consisted of the Swiss MS Registry, a recent prevalence estimation, and the Swiss mortality registry.

**Results:** The disease burden of MS in Switzerland in 2016 was 6,938 DALYs (95%-interval: 6,018-7,955), which corresponds to 97 DALYs per 100,000 adult inhabitants. Morbidity contributed 59% of the disease burden. While persons in an asymptomatic (EDSS-proxy 0) and mild (EDSS-proxy >0–3.5) disease stage represent 68.4% of the population, they make up 39.8% of the MS-specific morbidity. The remaining 60.2% of the MS-specific morbidity stems from the 31.6% of persons in a moderate (EDSS-proxy 4–6.5) or severe (EDSS-proxy ≥7) disease stage.

**Conclusions:** Morbidity has a larger influence on the disease burden of MS than mortality and is shared in a ratio of 2:3 between persons in an asymptomatic/mild and moderate/severe disease stage in Switzerland. Interventions to reduce severity worsening in combination with tailored, symptomatic treatments are important future paths to lower the disease burden of MS.

## Introduction

The disease burden is one of the most important indicators for the relevance of a certain condition, both from a societal as well as an individual perspective. The most commonly employed metric to describe the disease burden are Disability-Adjusted Life Years (DALYs). DALYs consist of two components. On the one hand, Years Lived with Disability (YLD) measure the burden in terms of the health-related quality of life (HRQoL) reduction of persons currently living with a disease. On the other hand, Years of Life Lost (YLL) quantify the effects of premature deaths accountable to the disease in terms of life-span reduction. Summing up these two components yields DALYS (1–3).

The Global Burden of Disease (GBD) 2016 Multiple Sclerosis Collaborators (2019) estimated that there are 2.2 million persons with Multiple Sclerosis (MS) worldwide and that 0.04% of all DALYs are due to MS. While these numbers might not seem extraordinarily high at first glance, they are nevertheless highly relevant because of the frequent onset of MS early in life (between 30 and 40 years) and the lack of a curative treatment. In fact, MS is the most common chronic, inflammatory, neurologic disease in young adults and therefore constitutes a comparatively high disease burden in younger age groups, with substantial reductions of health-related quality of life and significant health care need (4). Moreover, the prevalence in Western Europe and many other regions is relatively high and possibly rising. With an estimated prevalence of 174–187 cases per 100,000 inhabitants, Switzerland also represents a high prevalence country, but some estimates for other countries range even higher, exceeding 220 cases per 100,000 and with an increasing incidence (5, 6).

The disease burden of MS has been estimated previously for Switzerland in the context of larger, international consortia, which emphasized cross-country comparisons rather than more detailed estimates. Therefore, the results were either presented as summary measures pooling all disease stages or emphasized utility estimations (4, 7). More detailed, sub-group specific burden estimates are lacking. This knowledge gap is regrettable from a public health perspective as more detailed assessments reflecting the different disease-severity and age structure of the population of persons with MS (PwMS) are of high relevance to health policy and care providers. For example, measures of disease burden facilitate the evidence-based allocation of limited health care resources.

Our aim was two-fold: (i) to estimate the overall disease burden of MS in Switzerland and (ii) to identify the relative contributions of age-, sex-, and disease severity-specific subgroups to the overall disease burden of MS.

## Methods

### Study Population

For the present estimation, we used distributions of sex, age and disease severity obtained from the Swiss Multiple Sclerosis Registry (SMSR) on the basis of 1,412 PwMS (as of April 4, 2019; Table 1). The SMSR is an ongoing, prospective, observational, patient-centred study of adult PwMS in Switzerland. The information is directly obtained from PwMS through online participation or questionnaires on paper and is supplemented by a physician's confirmation of the diagnosis as well as further clinical information for a selected subpopulation. The structure of the SMSR was specifically developed to also include participants who are commonly not included in clinical studies as, for example, persons who were diagnosed recently, are highly disabled or treated by physician that do not actively participate in research (8, 9). In this manner, we were able to include the full spectrum of PwMS in Switzerland and minimize under-representations in the tails of the age-distribution. The SMSR study was approved by the ethics committee of the canton of Zurich (PB-2016-00894, BASEC 2019-01027) and written informed consent was obtained from all SMSR participants (8). All data were pseudonymised prior to analysis.

**Table 1:** Demographics and disease characteristics of the Swiss MS Registry sample.

	**Swiss MS registry**
Sample size	1,412
Sex female (%)	1,030 (72.9%)
Median age (IQR)	48.0 (38.0-56.0)
Current MS type	
RRMS (%)	1,007 (71.3%)
SPMS (%)	252 (17.8%)
PPMS (%)	153 (10.8%)
Disease severity	
Asymptomatic	145 (10.3%)
Mild	821 (58.1%)
Moderate	333 (23.6%)
Severe	113 (8.0%)
Median utility (IQR)	0.776 (0.637–0.916)
Median disease duration since diagnosis (IQR)	8.5 (3.1–16.5)
Median disease duration since first symptoms (IQR)	12.4 (5.2–21.0)
Median age at diagnosis (IQR)	36 (28–45)

*IQR, interquartile range; RRMS, relapsing-remitting MS; SPMS, secondary-progressive MS; PPMS, primary-progressive MS*.

The prevalence numbers needed for the morbidity component of the DALYs are based on the prevalence estimation of the study of Kaufmann et al. (5). Regarding the mortality component of the DALYs estimation, two different data sources were combined. The number of deaths, stratified by sex and age, were obtained in an aggregated form from the Swiss mortality registry (10). This registry includes persons for whom MS was recorded as main or secondary cause of death. Data on age- and sex-specific life expectancy in Switzerland were obtained from the Swiss federal statistical office (11, 12).

### Estimation of Disability-Adjusted Life Years (DALYs)

We followed the methodology that has previously been used by the GBD study called H-DALYs by Schroeder (1–3, 13). The DALYs were estimated by summing up YLD and YLL (DALYs = YLD + YLL) and no age weighting or discounting was considered. YLD were defined as

(1) $$\begin{array}{l} YLD=\underset{i,\;j,\;k}{\sum }\left(P_{ijk}*DW_{k}\right) \end{array}$$

and YLL as

(2) $$\begin{array}{l} YLL=\underset{i,\;j}{\sum }\left(D_{ij}*L_{ij}\right) \end{array}$$

with *i* and *j* being the indices for the age and sex groups and *k* the index for the disease severity groups. *P* stands for the prevalence, *DW* the disability weight, *D* the number of deaths and *L* the age- and sex-specific life expectancy.

Specifically, we worked with 17 age groups (18–19, 20–24,…, 90–94, and 95 and older), two sex groups (female and male) and four disease severity groups [asymptomatic (EDSS-proxy 0), mild (EDSS-proxy >0–3.5), moderate (EDSS-proxy 4–6.5), severe (EDSS-proxy 7–9.5)]. The EDSS-proxy values were estimated based on an algorithm by Barin et al. which uses self-reported mobility data (14). Furthermore, we added an asymptomatic category based on no mobility impairment and no problems in any of the five domains of the EQ-5D. The group-specific prevalences were obtained by using sex, age and disease severity distributions of the SMSR and by extrapolating the numbers from the 2016 prevalence estimation of Kaufmann et al. (5, 8, 9). The disability weights were used according to the GBD 2016 Multiple Sclerosis Collaborators (2019) (asymptomatic: 0 [0–0]; mild: 0.183 [0.124–0.253]; moderate: 0.463 [0.313–0.613]; severe: 0.719 [0.534–0.858]) (4). The number of deaths and the age- and sex-specific life expectancy were obtained from the Swiss mortality registry and the Swiss life expectancy table of 2016 by the Swiss Federal Office of Statistics (10–12).

### Statistical Analysis

We estimated YLD for every combination of sex, age and disease severity and YLL for every combination of sex and age according to Equations (1) and (2), respectively. Due to the uncertainty in the estimation of the involved parameters [prevalence (*P*), disability weight (*DW*) and number of deaths (*D*)], theses parameters were not considered in a fixed manner. Instead, each of them was presented as a range of plausible values. For the estimation, these ranges were treated as uniform distributions and one value sampled per parameter. To reasonably represent the existing uncertainty in the results, this estimation was repeated 1,000 times yielding a results distribution. For prevalence, the plausibility range was defined by the 95%-confidence interval previously reported in the estimation of Kaufmann et al. (5). The plausibility range of the disability weights was directly taken from the GBD 2016 Multiple Sclerosis Collaborators (2019) (4). The minimum and maximum of the number of deaths was given by the number of deaths if MS was considered as main cause of death only (lower limit), as well as main or secondary cause (upper limit) (10).

From these numbers, DALYs as well sub-group specific contributions to the disease burden of MS could be estimated. Due to sometimes skewed distributions, all burden measures are reported as medians (thus implying that subgroup specific burden estimates may not add up perfectly to the overall burden estimate).

As a sensitivity analysis, we re-estimated the disability weights of the four disease severity categories using empirical EQ-5D assessments from the SMSR using the French index value set and subtracting the utilities from 1 (4, 14–17).

All statistical analyses were performed using R, version 3.6.0 (18).

## Results

Population characteristics were obtained from 1,412 PwMS participating in the SMSR with a median age of 48 and a percentage of women of 72.9% (Table 1). Relapsing-remitting MS (RRMS) was the dominant disease course (71.3%), followed by secondary-progressive MS (SPMS) (17.8%) and primary-progressive MS (PPMS) (10.8%). The median age at diagnosis was 36 years, and time since diagnosis (median 8.5 years) and since first symptoms (median 12.4 years) were in the expected range.

Our estimation of the DALYs distribution of MS in Switzerland in 2016 yielded a median disease burden of 6,938 DALYs (95% interval: 6,018–7,955 DALYs). Setting this estimate into the context of the entire adult population of Switzerland in 2016 (7,127,446), we arrived at a median disease burden of MS of 97 DALYs per 100,000 adult inhabitants (95%-interval: 84–112 DALYs/100,000 inhabitants) (Figure A1). Looking at the sex-specific numbers, women—representing almost three quarters of individuals in the SMSR—contributed 67% of overall DALYS while men contributed 33% (Figures A2, A3). All data are displayed in Table 2.

**Table 2:** Disease burden of multiple sclerosis in Switzerland in 2016.

	**DALYs**	**YLD**	**YLL**
Absolute	6,938 (6,018–7,955)	4,129 (3,258–5,097)	2,811 (2,568–3,030)
Per 100,000	97 (84–112)	58 (46–72)	39 (36–43)
Women absolute	4,673 (4,039–5,404)	2,821 (2,208–3,486)	1,857 (1,656–2,036)
Women per 100,000	129 (112–149)	78 (61–96)	51 (46–56)
Men absolute	2,263 (1,953–2,588)	1,306 (1,043–1,603)	950 (813–1,090)
Men per 100,000	65 (56–74)	37 (30–46)	27 (23–31)

*The first column displays Disability-Adjusted Life Years (DALYs) and columns 2 and 3 the two components of it, years lived with disability (YLD) and years of life lost (YLL), respectively. The rows are always first displaying the absolute values and then the relative ones per 100,000 inhabitants. Global Burden of Disease study estimates are displayed in Table A1 (Supplementary Material)*.

**Figure 1:**
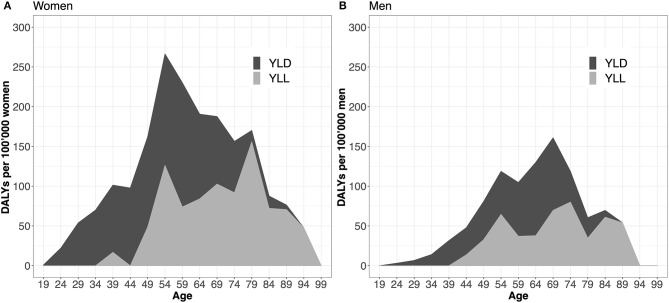
Years lived with disability (YLD) and years of life lost (YLL) per 100,000 inhabitants stratified by age and sex. Panel **(A)** displays the distribution of YLD and YLL components over the entire age spectrum for women, **(B)** for men. The two curves (YLL and YLD) are additive and summed up yield Disability-Adjusted Life Years (DALYs).

The individual contributions of YLD and YLL were 59 and 41%, respectively. Per 100,000, this yields a median YLD of 58/100,000 (95%-interval: 46–72/100,000) and a median YLL of 39/100,000 (36–43/100,000). Furthermore, we examined at the age- and sex-specific YLD and YLL patterns, shown in Figure 1. In both sexes, YLD are dominant before, YLL after the age of 70.

The distribution in the SMSR shows that 68.4% of the population are in an asymptomatic or mild disease stage (asymptomatic: 10.3%; mild: 58.1%) whereas 31.6% have a moderate to severe disability (moderate: 23.6%; severe: 8.0%) (Table 1). However, when examining the morbidity aspect of DALYs (= YLDs), PwMS in an asymptomatic or mild disease stage represent 39.8% of the MS-specific population morbidity (asymptomatic: 0 [0%]; mild: 1,644 [39.8%]). PwMS with a moderate to severe disability contribute 60.2% to the MS-specific morbidity (moderate: 1,646 [39.9%]; severe 840 [20.3%]).

Regarding the sensitivity analysis and estimating disability weights from the SMSR data, the results were all in a similar range compared to the relatively wide ones of the GBD 2016 Multiple Sclerosis Collaborators (group-wise disability weights: *asymptomatic:* SMSR interquartile range [IQR] 0–0, GBD 95%-confidence interval [95% CI] 0–0; *mild:* SMSR IQR 0.105–0.271, GBD 95% CI 0.124–0.253; *moderate:* SMSR IQR 0.276–0.452, GBD 95% CI 0.313–0.613; *severe:* SMSR IQR 0.478–0.730, GBD 95% CI 0.534–0.858) (4).

## Discussion

This study estimated the disease burden of multiple sclerosis in Switzerland in the year 2016 and investigated age- and sex-specific patterns. The median estimate was 97 DALYs per 100,000 inhabitants, with morbidity (YLD) being responsible for a share of 59% (58/100,000). Although representing 72.9% of the whole MS population, women contributed 2/3 of the overall disease burden of MS. The data showed that YLD were the driver before, YLL after the age of 70. While having a high population share of 68.4%, PwMS with an asymptomatic or mild disease severity make up about 39.8% of the morbidity-related, MS-specific disease burden.

Setting the number of DALYs of our study into context, they agree well with the estimate of the GBD study in 2016 for Switzerland (SMSR: 6,938 DALYs; GBD: 6,658 DALYs) (Table 2 and Table A1) (4). However, at closer inspection, some differences appear. The YLD components of our estimation is larger (SMSR: 4,129; GBD: 3,588) which can be explained by the fact that the prevalence estimation of the SMSR (considering multiple Swiss databases and a refined methodology) is higher (SMSR: ~15,200; GBD: ~13,950) and the GBD estimate is corrected for co-morbidities. In turn, the YLL component of the GBD estimate is larger (SMSR: 2,811; GBD: 3,070). This is surprising because the number of deaths considered in the GBD study (127) is at the lower end of our interval of possible number of deaths (120–183). A possible explanation for the differing assessments of YLL components might be that the GBD studies, which are based on multinational data, potentially assume that more deaths due to MS are occurring at younger ages, which would directly scale with the extended age-specific life expectancy (4).

MS plays a more important role regarding disease burden in Switzerland compared to other regions of the world. The burden of MS contributes 0.4% to the total DALYs in Switzerland, which is substantially more than the 0.04% worldwide, but comparable to Northern European countries (19). This can mainly be attributed to two causes. One is clearly the lower overall disease burden in Switzerland (CH: 24,063 DALYs/100,000 inhabitants; world: 32,711/100,000). Second, MS has a higher relative prevalence in Switzerland compared to other world regions, where the overall burden is subject to stronger influences of infectious diseases (4, 19).

MS is a substantial, though not one of the strongest drivers of disease burden in Switzerland. For example, rheumatoid arthritis makes up 0.32% of the total DALYs in Switzerland per year, diabetes mellitus (type I and II) 3.56%, or Alzheimer's disease/dementias 3.79% (19). However, MS bears important societal impacts, which are not represented by DALYs, for example reduced work capacity or higher health-care costs, due to its usually early onset between 30 and 40 and chronic nature (7).

A striking finding that, to our knowledge, has not been reported before is the distribution of the MS-specific morbidity in the severity subgroups compared to their population share. PwMS at an asymptomatic or mild disease stage make up 68.4% of the Swiss MS population (10.3, 58.1%, respectively), however, their share of the morbidity component—to which only the mild disease stage contributes—is estimated to be 39.8%. If sex- and age-specific utilities of persons in a mild disease stage are compared to the general population, small but consistently lower health utilities are present on average (between −0.02 and −0.08) (17). Therefore, it seems that this group's contribution to the overall disease burden is mainly driven by its size. Moreover, against the background of a comparatively small health utility difference between the general population and persons with mild MS, potential interventions such as novel disease modifying treatments are likely to resound more on a population than an individual level according to our analysis.

However, it also needs to be emphasized that population-level burden and utilities do not necessarily reflect the individual, real-world situation. The EDSS-based disease stage categorization inherits the limitations of being mainly gait-focused and somewhat subjective in certain areas (e.g., sensory function, bowel, and bladder function). As a consequence, symptoms like fatigue, cognitive disturbances, and depression are not well-reflected in this categorization, although they are frequent and highly influential on quality of life on an individual level (14). This shortcoming can be seen in the wide ranges of the disability weights within EDSS-based disease severity subgroups, particularly in the sensitivity analysis based on utility data from the SMSR. Future studies should also consider alternative disease stage definitions, for example based on self-reported health-related quality of life (e.g., measured by EQ-5D), to achieve more reliable group-specific disease burden estimates. Nonetheless, this recategorization is unlikely to affect the overall disease burden—compared to the disease severity specific burden—on a population level.

Independent of the above-mentioned short-comings, the subgroup-specific burden estimates are highly relevant for health-care policy stakeholders, since they may help to allocate financial resources by refining macro-allocation strategies. In particular, investments to reduce disease progression have the potential to positively affect the disease burden. For example, the expected demographic aging will likely lead to shifts toward higher disability in the MS population as the age average will increase. Treatments prolonging the transition to higher disability, which coincides with rising age and MS duration, could therefore act as an important lever to lower the overall disease burden of MS. At the same time, assessing opportunities to reduce or stabilize the morbidity in the entire spectrum, for example, improved symptomatic pharmacological and non-pharmacological treatment options or adjusted work possibilities, would not only reduce or keep the disease burden of MS stable but might also substantially influence the economic burden on a population level. Whether these findings are context-specific to Switzerland or valid for other European countries will have to be determined.

Our study has notable strengths since the estimations are based on several reliable and complementary data sources and a thorough methodology. However, also several limitations exist. Previous analyses of the Swiss MS Registry revealed a slight under-representation of older age groups and higher disability, with the potential to underestimate the burden. Nonetheless, we are confident that the impact of this underrepresentation of older age groups is small. In fact, a hypothetically missing group of 100 persons with a severe MS (disability weight: 0.719) would only increase the overall DALYs by 71.9. Furthermore, in the SMSR, only adult PwMS are considered, while the number of pediatric cases in Switzerland is unknown. Assuming a similar prevalence as reported by Marrie et al. (20), Switzerland would have about 100 cases of pediatric MS. Together with the generally higher utility, their impact on the population burden is likely to be small as well. Next, the use of an EDSS-proxy instead of a clinically assessed EDSS is expected to only marginally affect the estimates because categorizations and not exact EDSS values are used and a previous study showed a good accuracy of the EDSS-proxy (14). Another source of uncertainty concerns the mortality within the context of MS since the details of mortality recording vary substantially in different mortality registries. However, by including the range of MS being the main or a secondary cause of death, the uncertainty of our estimate should have been limited. All named limitations have been reduced by the resampling approach applied in this study by reflecting the uncertainty in the input parameters. As a final note, we would like to highlight that we estimated the MS-specific disease burden, which does not consider co-morbidities. Therefore, the disease burden in persons with MS—compared to the disease burden of MS—is likely still higher for two reasons. First, the co-morbidities alone can increase the burden. Second, interactions of the diseases might amplify the burden of specific symptoms. These patterns are more likely to be visible in older PwMS due to their higher rate of co-morbidities. However, the extent to which the population burden would be increased is unclear. A study of our group showed a limited direct influence of co-morbidities on the individual-level disease burden. Additionally, our sensitivity analysis, based on the quality of life of PwMS and therefore inherently also including co-morbidities, resulted in similar disability weights as the GBD study.

We conclude that the disease burden of MS in Switzerland is partitioned into 41% by mortality and 59% by morbidity. The latter is shared in a ratio of 2:3 by the high proportion of persons with asymptomatic or mild disease (68.4%) and the smaller (31.6%), but more impaired group of persons with moderate or severe disease disability. The further development of novel disease-modifying therapies aimed to attenuate progression in more advanced disease stages, might further increase the morbidity-associated burden share, but could at the same time also decrease the overall population level burden.

## Data Availability Statement

The datasets for this article are not publicly available because the authors do not have the permissions to share them. Requests to access the datasets should be directed to PD Dr. Viktor von Wyl, viktor.vonwyl@uzh.ch.

## Ethics Statement

The studies involving human participants were reviewed and approved by the Ethics committee of the canton of Zurich, Canton of Zurich, Switzerland. The patients/participants provided their written informed consent to participate in this study.

## Author Contributions

All authors contributed to the study conception and data collection. Design and statistical analysis were performed and the first draft of the manuscript written by MP, VW, and MK. All authors commented on the manuscript and read and approved the final version.

### Conflict of Interest

AS received speaker honoraria and/or travel compensation for activities with Almirall Hermal GmbH, Biogen, Merck, Novartis, Roche, and Sanofi Genzyme, none related to this work. CG: The Department of Neurology, Regional Hospital Lugano (EOC), Lugano, Switzerland, receives financial support from Teva, Merck Serono, Biogen, Genzyme, Roche, Celgene, Bayer, and Novartis. The submitted work is not related to these agreements. CP has received travel grants or participated to advisory boards for Merck, Biogen IDEC, Roche, Novartis, Genzyme and Celgene, all not related to this manuscript. CK has received honoraria for lectures as well as research support from Biogen, Novartis, Almirall, Bayer Schweiz AG, Eli Lilly, Teva, Merck, Sanofi Genzyme, Roche, Celgene and the Swiss MS Society (SMSG). JK's institution (University Hospital Basel) received and used exclusively for research support consulting fees from Biogen, Novartis, Protagen AG, Roche, and Teva; speaker fees from Biogen, Genzyme, Novartis, Roche, and the Swiss Multiple Sclerosis Society; travel expenses from Merck Serono, Novartis, and Roche; grants from Bayer AG, Biogen, the ECTRIMS Research Fellowship Programme, Genzyme, Merck, Novartis, Roche, the Swiss Multiple Sclerosis Society, the Swiss National Research Foundation (320030_160221), and the University of Basel. PC has received honoraria for speaking at scientific meetings, serving at scientific advisory boards and consulting activities from: Abbvie, Actelion, Almirall, Bayer-Schering, Biogen Idec, EISAI, Genzyme, Lundbeck, Merck Serono, Novartis, Pfizer, Teva, and Sanofi-Aventis; his research is also supported by the Swiss Multiple Sclerosis Society, the Swiss National Research Foundation and the SOFIA Foundation. SM received honoraria for travel, honoraria for lectures/consulting, and/or grants for studies from Almirall, Biogen, Celgene, Novartis, Teva, Merck Serono, Genzyme, Roche, and Bayer Schweiz AG. SS has received consulting and speaker fees as well as travel grants from Bayer, Biogen, Celgene, Merck, Novartis, Roche, Sanofi Genzyme and Teva. He has received unrestricted research grants from Novartis and Sanofi Genzyme. None related to the current work. The remaining authors declare that the research was conducted in the absence of any commercial or financial relationships that could be construed as a potential conflict of interest.
