# Supplementary material for: 60/30: 60% of the Morbidity-Associated Multiple Sclerosis Disease Burden Comes From the 30% of Persons With Higher Impairments
Source: Front Neurol. 2020 Mar 6;11:156. doi: 10.3389/fneur.2020.00156 (PMC7068809; doi:10.3389/fneur.2020.00156)
Supplement: Supplementary file 1 [file Table_1.docx]

Supplementary Material

Table A 1: Disease burden of multiple sclerosis in Switzerland in 2016 according to the Global Burden of Disease study (GBD).

|  | DALYs | YLD | YLL |
| --- | --- | --- | --- |
| Absolute | 6658 (5088-8463) | 3588 (2481-4688) | 3070 (2094-4489) |
| Per 100'000 | 79 (61-101) | 43 (30-56) | 37 (25-54) |
| Women absolute | 4628 (3389-6072) | 2572 (1802-3383) | 2056 (1267-3341) |
| Women per 100'000 | 109 (80-144) | 61 (43-80) | 49 (30-79) |
| Men absolute | 2030 (1515-2650) | 1016 (685-1372) | 1014 (650-1514) |
| Men per 100'000 | 49 (37-64) | 24 (17-33) | 24 (16-36) |

The first column displays Disability-Adjusted Life Years (DALYs) and columns 2 and 3 the two components of it, years lived with disability (YLD) and years of life lost (YLL), respectively. The rows are always first displaying the absolute values and then the relative ones per 100,000 inhabitants. Remark: The relative numbers in the GBD study (per 100,000) were calculated in the entire Swiss population, the numbers of this study only in the adult Swiss population (cf. Table 2), which explains the differences. Institute for Health Metrics and Evaluation (IHME), 2018. GBD Compare Data visualization. Seattle, WA IHME, Univ. Washington.


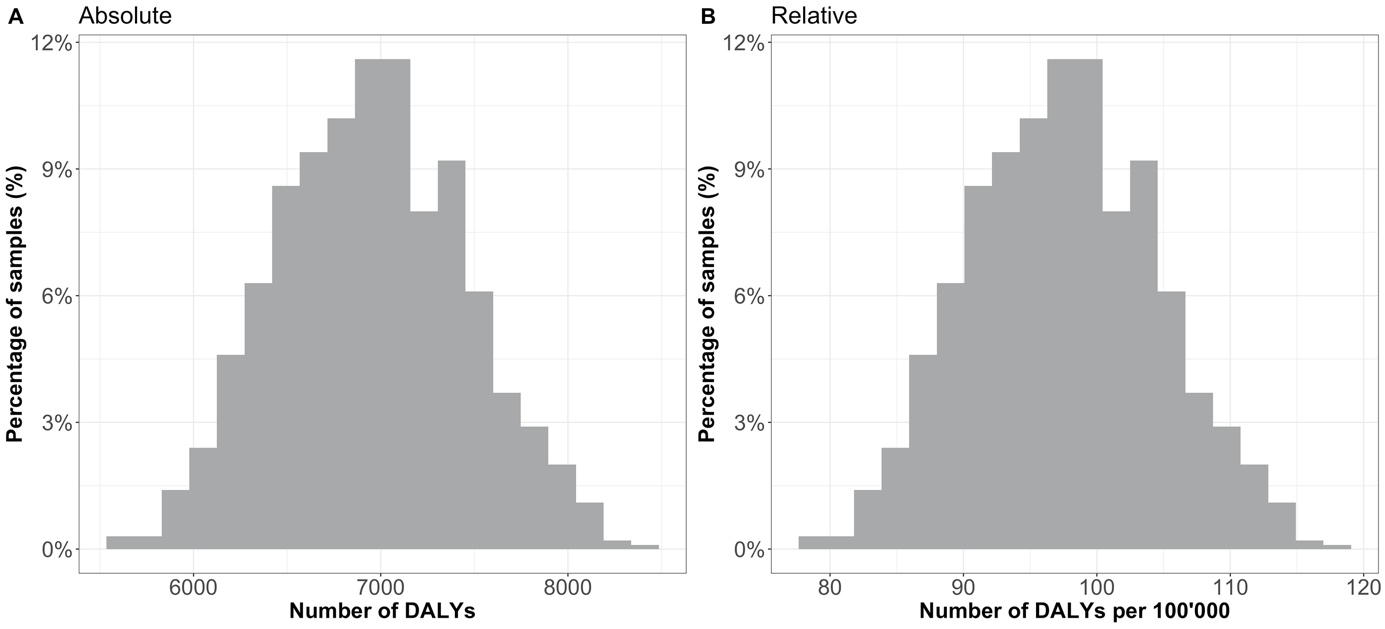


Figure A 1: Distribution resulting from estimating Disability-Adjusted Life Years (DALYs) with resampling. Figure A displays the absolute DALYs while Figure B shows the relative DALYs per 100,000 inhabitants. The percentage of samples refers to the percentage of the 1000 resamples that resulted in the corresponding number of DALYs.


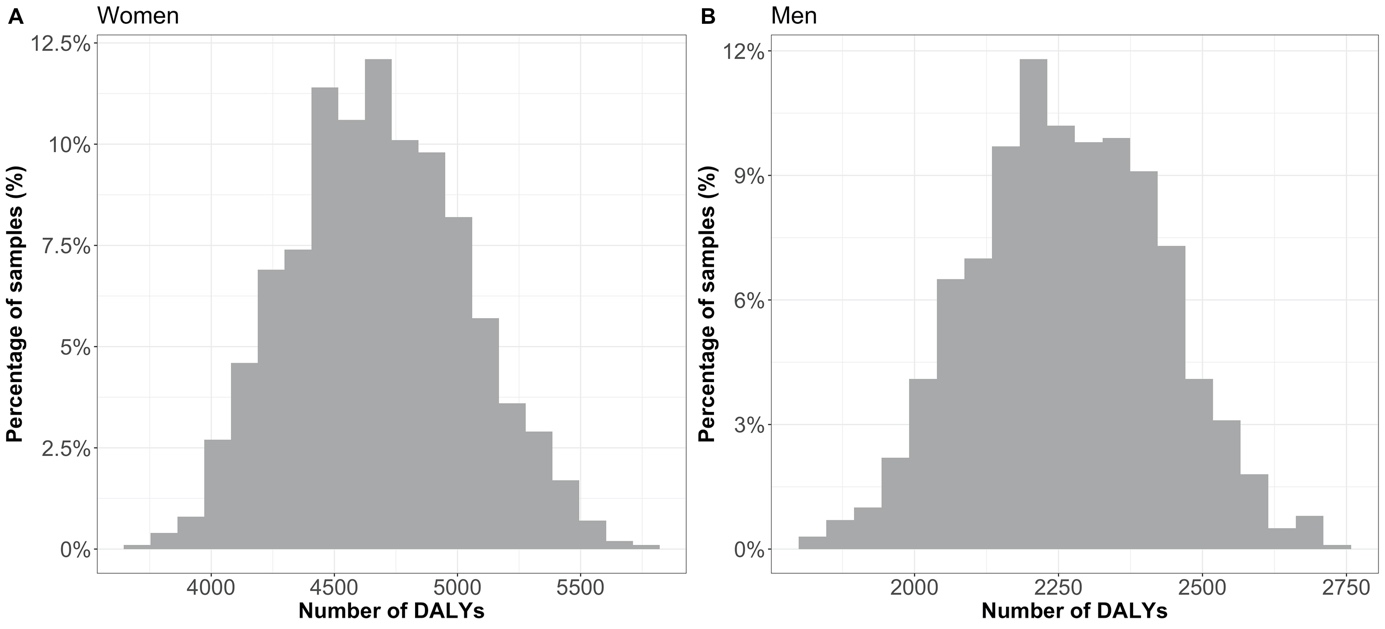


Figure A 2: Distribution resulting from estimating Disability-Adjusted Life Years (DALYs) with resampling for women and men individually. Figure A displays the absolute DALYs for women and Figure B for men. The percentage of samples refers to the percentage of the 1000 resamples that resulted in the corresponding number of DALYs.


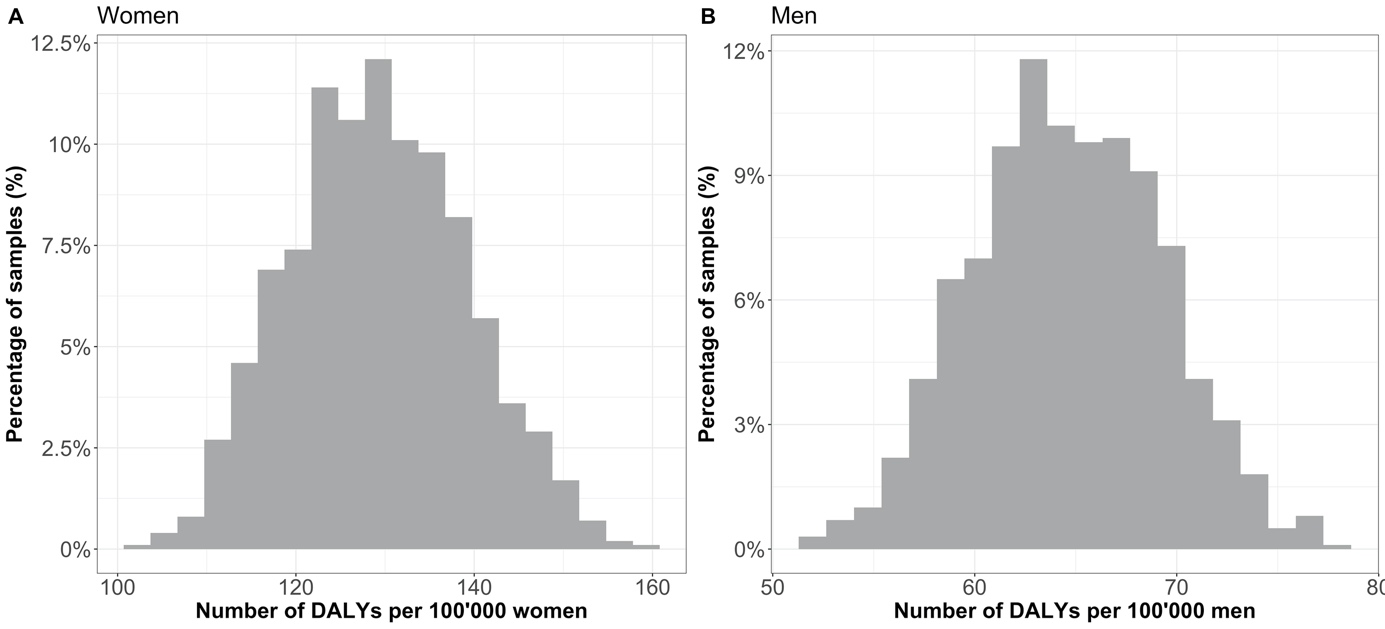


Figure A 3: Distribution resulting from estimating Disability-Adjusted Life Years (DALYs) with resampling for women and men individually per 100’000 inhabitants. Figure A displays the DALYs per 100’000 inhabitants for women and Figure B for men. The percentage of samples refers to the percentage of the 1000 resamples that resulted in the corresponding number of DALYs.
